# [2-(Piperidin-1-yl)ethyl­amine]dithio­cyanato­zinc(II)

**DOI:** 10.1107/S1600536810007300

**Published:** 2010-03-03

**Authors:** Chen-Yi Wang, Ai-Fei Ke, Xiang Wu

**Affiliations:** a Department of Chemistry, Huzhou University, Huzhou 313000, People’s Republic of China

## Abstract

In the mononuclear title compound, [Zn(NCS)_2_(C_7_H_16_N_2_)], the Zn^II^ atom is four-coordinated by two N atoms of the chelating 2-(piperidin-1-yl)ethyl­amine ligand and two N atoms from two thio­cyanate ligands in a distorted tetra­hedral geometry. In the crystal structure, mol­ecules are linked through inter­molecular N—H⋯S hydrogen bonds, forming chains along the *b* axis.

## Related literature

For related structures, see: Wang *et al.* (2009*a* ▶,*b* ▶); Wang (2009 ▶). For bond-length and angle data, see: Cameron *et al.* (1998 ▶); Hong (2007 ▶).
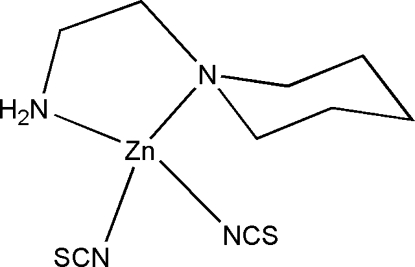


## Experimental

### 

#### Crystal data

[Zn(NCS)_2_(C_7_H_16_N_2_)]
*M* *_r_* = 309.75
Monoclinic, 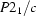

*a* = 9.561 (2) Å
*b* = 10.310 (2) Å
*c* = 14.398 (3) Å
β = 97.367 (3)°
*V* = 1407.6 (5) Å^3^
*Z* = 4
Mo *K*α radiation
μ = 2.02 mm^−1^
*T* = 298 K
0.20 × 0.20 × 0.18 mm

#### Data collection

Bruker SMART CCD area-detector diffractometer
Absorption correction: multi-scan (*SADABS*; Sheldrick, 1996 ▶) *T*_min_ = 0.688, *T*_max_ = 0.712
7615 measured reflections
3029 independent reflections
2196 reflections with *I* > 2σ(*I*)
*R*_int_ = 0.028

#### Refinement

*R*[*F* ^2^ > 2σ(*F* ^2^)] = 0.035
*wR*(*F* ^2^) = 0.095
*S* = 1.04
3029 reflections
145 parameters
H-atom parameters constrained
Δρ_max_ = 0.32 e Å^−3^
Δρ_min_ = −0.39 e Å^−3^

### 

Data collection: *SMART* (Bruker, 1998 ▶); cell refinement: *SAINT* (Bruker, 1998 ▶); data reduction: *SAINT*; program(s) used to solve structure: *SHELXS97* (Sheldrick, 2008 ▶); program(s) used to refine structure: *SHELXL97* (Sheldrick, 2008 ▶); molecular graphics: *SHELXTL* (Sheldrick, 2008 ▶); software used to prepare material for publication: *SHELXTL*.

## Supplementary Material

Crystal structure: contains datablocks global, I. DOI: 10.1107/S1600536810007300/ci5046sup1.cif

Structure factors: contains datablocks I. DOI: 10.1107/S1600536810007300/ci5046Isup2.hkl

Additional supplementary materials:  crystallographic information; 3D view; checkCIF report

## Figures and Tables

**Table 1:** Hydrogen-bond geometry (Å, °)

*D*—H⋯*A*	*D*—H	H⋯*A*	*D*⋯*A*	*D*—H⋯*A*
N1—H1*A*⋯S1^i^	0.90	2.65	3.523 (3)	165
N1—H1*B*⋯S2^ii^	0.90	2.71	3.509 (3)	148

Symmetry codes: (i) 
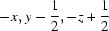
; (ii) 
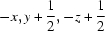
.

**Fig. 1.**
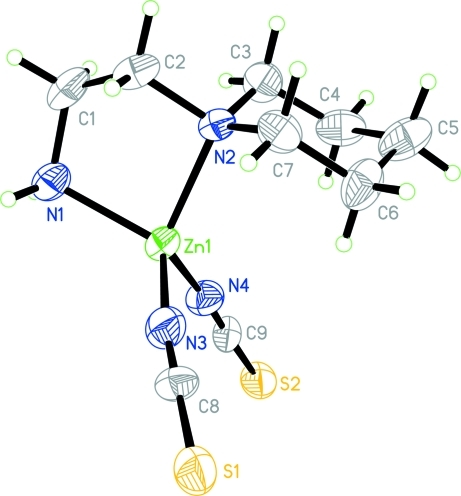
The molecular structure of the title compound, showing the atom-numbering scheme. Displacement ellipsoids are drawn at the 30% probability level.

**Fig. 2.**
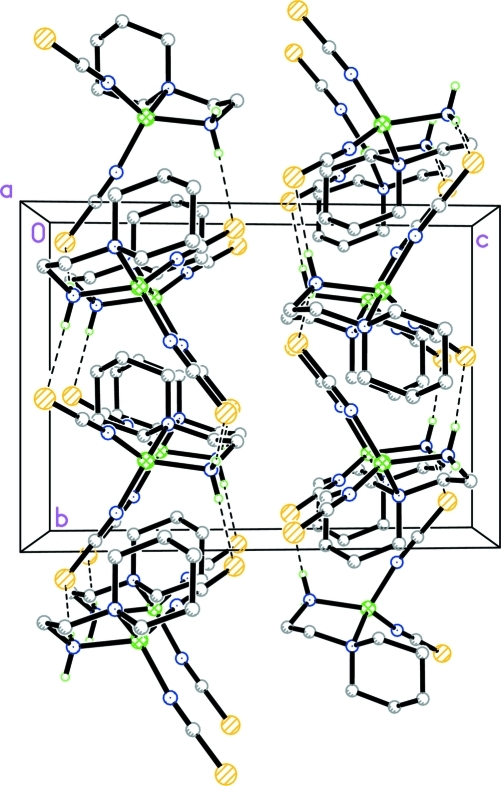
The molecular packing of the title compound, viewed along the a axis. Intermolecular N—H···S hydrogen bonds are shown as dashed lines.

## supplementary crystallographic information

### Comment

As part of our investigations into novel urease inhibitors (Wang *et al.*, 2009a,b; Wang, 2009), we have synthesized the title compound, a new Zn^II^ complex, and its crystal structure is reported here.

The Zn^II^ atom in the complex is chelated by the two N atoms of 2-piperidin-1-ylethylamine ligand and two N atoms from two thiocyanate ligands, giving a distorted tetrahedral geometry (Fig. 1). The coordinate bond lengths and angles are typical and are comparable with those observed in other related zinc(II) complexes (Cameron *et al.*, 1998; Hong, 2007).

In the crystal structure, molecules are linked through intermolecular N—H···S hydrogen bonds (Table 1), forming chains running along the *b* axis (Fig. 2).

### Experimental

2-Piperidin-1-ylethylamine (1.0 mmol, 128 mg), ammonium thiocyanate (1.0 mmol, 76 mg), and Zn(NO_3_)_2_.6H_2_O (1.0 mmol, 290 mg) were dissolved in MeOH (30 ml). The mixture was stirred at room temperature for 10 min to give a clear colourless solution. After keeping the solution in air for a week, colourless block-shaped crystals were formed at the bottom of the vessel.

### Refinement

H atoms were placed in geometrically idealized positions and constrained to ride on their parent atoms, with C–H distances of 0.97 Å, N–H distances of 0.90 Å, and with U_iso_(H) set at 1.2U_eq_(C,N).

### Crystal data

**Table:**

[Zn(NCS)_2_(C_7_H_16_N_2_)]	*F*(000) = 640
*M**_r_* = 309.75	*D*_x_ = 1.462 Mg m^−^^3^
Monoclinic, *P*2_1_/*c*	Mo *K*α radiation, λ = 0.71073 Å
Hall symbol: -P 2ybc	Cell parameters from 2403 reflections
*a* = 9.561 (2) Å	θ = 2.4–25.0°
*b* = 10.310 (2) Å	µ = 2.02 mm^−^^1^
*c* = 14.398 (3) Å	*T* = 298 K
β = 97.367 (3)°	Block, colourless
*V* = 1407.6 (5) Å^3^	0.20 × 0.20 × 0.18 mm
*Z* = 4	

### Data collection

**Table:**

Bruker SMART CCD area-detector diffractometer	3029 independent reflections
Radiation source: fine-focus sealed tube	2196 reflections with *I* > 2σ(*I*)
graphite	*R*_int_ = 0.028
ω scan	θ_max_ = 27.0°, θ_min_ = 2.4°
Absorption correction: multi-scan (*SADABS*; Sheldrick, 1996)	*h* = −12→11
*T*_min_ = 0.688, *T*_max_ = 0.712	*k* = −13→13
7615 measured reflections	*l* = −9→18

### Refinement

**Table:**

Refinement on *F*^2^	Primary atom site location: structure-invariant direct methods
Least-squares matrix: full	Secondary atom site location: difference Fourier map
*R*[*F*^2^ > 2σ(*F*^2^)] = 0.035	Hydrogen site location: inferred from neighbouring sites
*wR*(*F*^2^) = 0.095	H-atom parameters constrained
*S* = 1.04	*w* = 1/[σ^2^(*F*_o_^2^) + (0.0475*P*)^2^ + 0.1775*P*] where *P* = (*F*_o_^2^ + 2*F*_c_^2^)/3
3029 reflections	(Δ/σ)_max_ = 0.001
145 parameters	Δρ_max_ = 0.32 e Å^−^^3^
0 restraints	Δρ_min_ = −0.38 e Å^−^^3^

### Special details

**Table:**

Geometry. All esds (except the esd in the dihedral angle between two l.s. planes) are estimated using the full covariance matrix. The cell esds are taken into account individually in the estimation of esds in distances, angles and torsion angles; correlations between esds in cell parameters are only used when they are defined by crystal symmetry. An approximate (isotropic) treatment of cell esds is used for estimating esds involving l.s. planes.
Refinement. Refinement of *F*^2^ against ALL reflections. The weighted *R*-factor wR and goodness of fit *S* are based on *F*^2^, conventional *R*-factors *R* are based on *F*, with *F* set to zero for negative *F*^2^. The threshold expression of *F*^2^ > σ(*F*^2^) is used only for calculating *R*-factors(gt) etc. and is not relevant to the choice of reflections for refinement. *R*-factors based on *F*^2^ are statistically about twice as large as those based on *F*, and *R*- factors based on ALL data will be even larger.

### Fractional atomic coordinates and isotropic or equivalent isotropic displacement parameters (Å^2^)

**Table:**

	*x*	*y*	*z*	*U*_iso_*/*U*_eq_	
Zn1	0.10783 (3)	0.24465 (3)	0.24635 (2)	0.05057 (13)	
S1	0.28910 (10)	0.60126 (8)	0.42530 (6)	0.0791 (3)	
S2	−0.19929 (8)	0.06124 (9)	0.43790 (6)	0.0750 (3)	
N1	0.0345 (3)	0.2689 (2)	0.10961 (16)	0.0620 (6)	
H1A	−0.0556	0.2418	0.0979	0.074*	
H1B	0.0382	0.3530	0.0936	0.074*	
N2	0.2756 (2)	0.1410 (2)	0.20451 (14)	0.0495 (5)	
N3	0.1803 (3)	0.4000 (2)	0.31175 (18)	0.0706 (7)	
N4	−0.0197 (3)	0.1600 (3)	0.32020 (17)	0.0676 (6)	
C1	0.1265 (4)	0.1900 (4)	0.0560 (2)	0.0756 (9)	
H1C	0.1262	0.2262	−0.0062	0.091*	
H1D	0.0907	0.1020	0.0495	0.091*	
C2	0.2741 (3)	0.1887 (3)	0.1060 (2)	0.0671 (8)	
H2A	0.3131	0.2756	0.1068	0.081*	
H2B	0.3325	0.1328	0.0728	0.081*	
C3	0.2624 (3)	−0.0025 (3)	0.2047 (2)	0.0678 (8)	
H3A	0.3371	−0.0402	0.1738	0.081*	
H3B	0.1729	−0.0272	0.1695	0.081*	
C4	0.2706 (4)	−0.0557 (3)	0.3028 (2)	0.0791 (9)	
H4A	0.2666	−0.1497	0.3001	0.095*	
H4B	0.1900	−0.0255	0.3313	0.095*	
C5	0.4045 (4)	−0.0145 (4)	0.3625 (2)	0.0883 (11)	
H5A	0.4031	−0.0439	0.4264	0.106*	
H5B	0.4853	−0.0533	0.3388	0.106*	
C6	0.4168 (3)	0.1305 (4)	0.3608 (2)	0.0826 (10)	
H6A	0.5048	0.1568	0.3970	0.099*	
H6B	0.3400	0.1689	0.3892	0.099*	
C7	0.4124 (3)	0.1786 (3)	0.2614 (2)	0.0691 (8)	
H7A	0.4222	0.2723	0.2615	0.083*	
H7B	0.4905	0.1418	0.2334	0.083*	
C8	0.2262 (3)	0.4841 (3)	0.35825 (19)	0.0552 (7)	
C9	−0.0958 (3)	0.1204 (3)	0.36869 (18)	0.0499 (6)	

### Atomic displacement parameters (Å^2^)

**Table:**

	*U*^11^	*U*^22^	*U*^33^	*U*^12^	*U*^13^	*U*^23^
Zn1	0.0569 (2)	0.0542 (2)	0.04129 (19)	0.00372 (13)	0.00884 (14)	−0.00022 (13)
S1	0.0986 (6)	0.0605 (5)	0.0736 (5)	−0.0117 (4)	−0.0061 (5)	−0.0036 (4)
S2	0.0604 (4)	0.0944 (6)	0.0750 (5)	−0.0167 (4)	0.0277 (4)	−0.0161 (4)
N1	0.0697 (15)	0.0669 (16)	0.0479 (13)	0.0098 (11)	0.0013 (12)	0.0075 (11)
N2	0.0570 (12)	0.0496 (12)	0.0432 (11)	0.0032 (10)	0.0111 (10)	0.0058 (9)
N3	0.101 (2)	0.0521 (15)	0.0585 (15)	−0.0003 (13)	0.0080 (13)	−0.0040 (12)
N4	0.0633 (14)	0.0833 (18)	0.0583 (14)	−0.0052 (13)	0.0162 (12)	0.0001 (13)
C1	0.107 (3)	0.080 (2)	0.0385 (15)	0.022 (2)	0.0079 (16)	0.0045 (15)
C2	0.080 (2)	0.075 (2)	0.0511 (17)	0.0150 (17)	0.0243 (15)	0.0135 (15)
C3	0.082 (2)	0.0526 (17)	0.0687 (19)	0.0049 (15)	0.0069 (16)	0.0014 (14)
C4	0.091 (2)	0.063 (2)	0.087 (2)	0.0138 (17)	0.0214 (19)	0.0278 (18)
C5	0.087 (2)	0.113 (3)	0.066 (2)	0.039 (2)	0.0130 (19)	0.028 (2)
C6	0.0624 (18)	0.118 (3)	0.063 (2)	0.0176 (19)	−0.0094 (15)	−0.003 (2)
C7	0.0511 (16)	0.073 (2)	0.084 (2)	−0.0015 (14)	0.0122 (16)	0.0011 (17)
C8	0.0658 (17)	0.0499 (16)	0.0513 (16)	0.0069 (13)	0.0126 (13)	0.0116 (13)
C9	0.0413 (13)	0.0584 (16)	0.0494 (15)	0.0024 (11)	0.0031 (11)	−0.0129 (12)

### Geometric parameters (Å, °)

**Table:**

Zn1—N4	1.927 (3)	C2—H2A	0.97
Zn1—N3	1.940 (3)	C2—H2B	0.97
Zn1—N1	2.019 (2)	C3—C4	1.508 (4)
Zn1—N2	2.080 (2)	C3—H3A	0.97
S1—C8	1.614 (3)	C3—H3B	0.97
S2—C9	1.611 (3)	C4—C5	1.509 (5)
N1—C1	1.485 (4)	C4—H4A	0.97
N1—H1A	0.90	C4—H4B	0.97
N1—H1B	0.90	C5—C6	1.500 (5)
N2—C3	1.485 (3)	C5—H5A	0.97
N2—C2	1.500 (3)	C5—H5B	0.97
N2—C7	1.503 (3)	C6—C7	1.510 (5)
N3—C8	1.148 (3)	C6—H6A	0.97
N4—C9	1.146 (3)	C6—H6B	0.97
C1—C2	1.500 (4)	C7—H7A	0.97
C1—H1C	0.97	C7—H7B	0.97
C1—H1D	0.97		
			
N4—Zn1—N3	108.54 (11)	N2—C3—C4	111.7 (3)
N4—Zn1—N1	115.39 (10)	N2—C3—H3A	109.3
N3—Zn1—N1	115.40 (10)	C4—C3—H3A	109.3
N4—Zn1—N2	119.57 (10)	N2—C3—H3B	109.3
N3—Zn1—N2	108.90 (10)	C4—C3—H3B	109.3
N1—Zn1—N2	88.02 (9)	H3A—C3—H3B	107.9
C1—N1—Zn1	106.51 (17)	C3—C4—C5	111.7 (3)
C1—N1—H1A	110.4	C3—C4—H4A	109.3
Zn1—N1—H1A	110.4	C5—C4—H4A	109.3
C1—N1—H1B	110.4	C3—C4—H4B	109.3
Zn1—N1—H1B	110.4	C5—C4—H4B	109.3
H1A—N1—H1B	108.6	H4A—C4—H4B	107.9
C3—N2—C2	109.8 (2)	C6—C5—C4	109.5 (3)
C3—N2—C7	108.9 (2)	C6—C5—H5A	109.8
C2—N2—C7	109.4 (2)	C4—C5—H5A	109.8
C3—N2—Zn1	116.27 (17)	C6—C5—H5B	109.8
C2—N2—Zn1	101.04 (16)	C4—C5—H5B	109.8
C7—N2—Zn1	111.06 (17)	H5A—C5—H5B	108.2
C8—N3—Zn1	173.1 (2)	C5—C6—C7	110.5 (3)
C9—N4—Zn1	173.6 (3)	C5—C6—H6A	109.5
N1—C1—C2	109.8 (3)	C7—C6—H6A	109.5
N1—C1—H1C	109.7	C5—C6—H6B	109.5
C2—C1—H1C	109.7	C7—C6—H6B	109.5
N1—C1—H1D	109.7	H6A—C6—H6B	108.1
C2—C1—H1D	109.7	N2—C7—C6	110.4 (2)
H1C—C1—H1D	108.2	N2—C7—H7A	109.6
C1—C2—N2	110.5 (2)	C6—C7—H7A	109.6
C1—C2—H2A	109.5	N2—C7—H7B	109.6
N2—C2—H2A	109.5	C6—C7—H7B	109.6
C1—C2—H2B	109.5	H7A—C7—H7B	108.1
N2—C2—H2B	109.5	N3—C8—S1	178.9 (3)
H2A—C2—H2B	108.1	N4—C9—S2	178.2 (3)

### Hydrogen-bond geometry (Å, °)

**Table:**

*D*—H···*A*	*D*—H	H···*A*	*D*···*A*	*D*—H···*A*
N1—H1A···S1^i^	0.90	2.65	3.523 (3)	165
N1—H1B···S2^ii^	0.90	2.71	3.509 (3)	148

Symmetry codes: (i) −*x*, *y*−1/2, −*z*+1/2; (ii) −*x*, *y*+1/2, −*z*+1/2.
